# Auditory Emotional Prosody Perception Using Pseudo‐Speech Stimuli in Native and Non‐Native Listeners

**DOI:** 10.1002/brb3.70475

**Published:** 2025-04-07

**Authors:** Emre Gürses, Sıdıka Cesur, Vinaya Manchaiah

**Affiliations:** ^1^ Department of Audiology, Faculty of Health Sciences Hacettepe University Ankara Turkey; ^2^ Virtual Hearing Lab, Collaborative Initiative Between University of Colorado School of Medicine, Aurora, Colorado, USA, and University of Pretoria Pretoria South Africa; ^3^ Department of Audiology Faculty of Health Sciences İstanbul Medeniyet University İstanbul Turkey; ^4^ Department of Otolaryngology–Head and Neck Surgery University of Colorado School of Medicine Aurora Colorado USA; ^5^ UCHealth Hearing and Balance University of Colorado Hospital Aurora Colorado USA; ^6^ Department of Speech‐Language Pathology and Audiology University of Pretoria Pretoria South Africa; ^7^ Department of Speech and Hearing, School of Allied Health Sciences Manipal Academy of Higher Education Manipal India

**Keywords:** auditory perception, non‐native listeners, prosody perception, pseudo‐sentences, speech processing

## Abstract

**Objective:**

To assess emotional recognition ability using corpora of emotions conveyed through nonlinguistic pseudo‐sentences in native and non‐native listeners (Turkish and English).

**Methods:**

A cross‐sectional design was employed, including a total of 60 young adults (aged 18–25 years). Of these, 30 were American English‐speaking participants with no knowledge of Turkish, while the remaining 30 were age‐, sex‐, and education‐matched Turkish participants. Emotional recognition scores and reaction times were assessed following audiological measurements using a one‐interval, five‐alternative forced choice method. A hundred stimuli recorded by Turkish speakers were presented, including 5 emotions × 2 speakers × 10 pseudo‐sentences. The emotions tested were “neutral,” “happy,” “angry,” “surprised,” and “panicked.”

**Results:**

No statistically significant differences exist between the groups' recognition of “neutral” and “angry” emotions. However, significant differences were observed in the recognition of happy, surprised, panicked and the mean scores of the emotions. Reaction times showed that nonmeaningful pseudo‐sentences elicited similar listening efforts between native and non‐native listeners.

**Conclusion:**

Overall results suggest that while there may be recognizable vocal cues irrespective of languages for expressing angry and neutral emotions, this does not apply to all emotions. These results point to the fact that the type of test materials may play an important role when measuring emotional recognition among different cultures using auditory stimuli. In terms of reaction time results, pseudo‐sentences could be used for cross‐language auditory emotion recognition, however with certain emotions.

## Graphical abstract

Anger and neutral appear to be universally recognized, whereas happiness, surprise, and panic depend more on linguistic familiarity.

Pseudo‐sentences can be a valuable tool for assessing universal emotional perception, but recognition performance varies depending on the emotion type.

Reaction time measures suggest that pseudo‐sentences impose similar cognitive loads across languages, supporting their potential use in universal prosody test batteries.

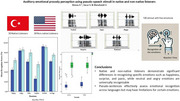

## 1 Introduction

Linguistic features play a key role in recognizing emotional content, but nonlinguistic cues, such as laughter, also contribute to decoding meaning. Laughter, a nonverbal vocalization with minimal articulatory movements and modified breathing, can convey unspoken intentions (Szameitat et al. 2009). Research suggests that emotional prosody is recognized similarly in speech and pseudowords, and even a single vowel sound can effectively communicate emotions (Belin et al. 2008). Paul Ekman identified universal facial expressions for emotions like enjoyment, sadness, and anger, raising the question of whether auditory emotional cues transcend language and culture. Various hypotheses have been suggested about cultural and universal emotion recognition. While evolutionary theory suggests prosodic cues are universally shared due to their adaptive significance (Bryant 2021), the cultural proximity hypothesis argues that emotion recognition is more accurate among culturally similar groups (Elfenbein and Nalini 2003). However, the interplay of verbal and nonverbal cues in cross‐cultural vocal emotion recognition remains unclear.

Our previous research explored sex‐related patterns in emotional prosody production and perception (Ertürk et al. 2024). The findings revealed no significant sex differences in emotional prosody perception. Similarly, Ćwiek et al. (2021) conducted an online study across 28 languages from 12 language families and found that nonlinguistic emotional vocalizations were recognized with above‐chance accuracy. Based on their results, the authors suggested that these vocalizations are not significantly influenced by cross‐cultural differences, making them promising candidates for a universal auditory prosody test battery.

The stimuli in Ćwiek et al.’s study were recorded by English‐speaking individuals, and familiarity with English as a second language was noted among many non‐native listeners. Given the widespread global exposure to English‐language media, further research involving less familiar languages is necessary to better assess the crosslinguistic and cross‐cultural effects of nonlinguistic vocal stimuli.

The present study aimed to assess emotional recognition abilities using an emotion corpus composed of nonlinguistic pseudo‐sentences across two distinct linguistic and cultural groups. The stimuli consisted of nonsense words spoken by non‐English speakers (Turkish) in both languages. Participants were selected from two language groups: English, an Indo‐European (Germanic) language, and Turkish, a Turkic language. This selection allowed for the comparison of crosslinguistic and cross‐cultural differences in emotional prosody perception.

Exposure to foreign languages or linguistic challenges introduces varying degrees of acoustic mismatch, requiring different levels of cognitive effort depending on the task's acoustic, linguistic, and cognitive demands (Peelle 2018). Reaction time is often used as an indicator of processing time, reflecting the cognitive load or listening effort involved in such tasks (Kılıç et al. 2022). In this study, reaction times for emotional responses were also assessed to understand the impact of pseudo‐sentences on listening effort among participants with diverse linguistic backgrounds (Giuliani et al. 2021). However, intrinsic and extrinsic factors, such as age, gender, intelligence, anxiety, or methodological task demands, can potentially influence the results (Paraskevopoulou et al. 2021). To minimize these influences, we included participants matched for sex, age, and education level.

## 2 Method

The study used a cross‐sectional design and involved comparing two different cultures (Turkey and the United States) in terms of five different emotions. The study was approved by the ethics committee at Hacettepe University in Turkey (May 31, 2022, GO 22/545) and the institutional review board at Lamar University in the United States (IRB‐FY22‐3). Ethical approval was given in two stages. In the present paper, the results of the first stage were presented. Our study was conducted according to the principles expressed in the Declaration of Helsinki. All participants completed the written informed consent prior to participating in the study.

### 2.1 Participants

A sample of 60 young adults (18–25 years) was included in the study. Of these, 30 participants were American English‐speaking participants who had no knowledge of Turkish, and the remaining 30 participants were age‐, sex‐, and education‐matched Turkish participants. Study participants were recruited by posting study advertisements on various platforms, including the university online mailing lists as well as social media pages.

The inclusion criteria were as follows: Native English or Turkish speakers, familiarity with the use of a computer, bilaterally normal hearing at all octave frequencies ≤ 20 dB HL as tested with pure tone audiometry, no active middle ear infection verified with tympanometry, and speech discrimination scores of at least 92% as tested in quiet using 25 phonetically balanced words in their native language. Second language (L2) Turkish learning for the native American English group and a history of major neurological or psychiatric disorders for both groups were exclusion criteria.

### 2.2 Procedure

The data collection consisted of audiological evaluations, emotional prosody recognition assessment as well as motor response and reaction time assessment, as explained below. All tests were carried out in a sound‐attenuated booth via supraural headphones.

#### 2.2.1 Audiological Evaluations

PTA was performed in a sound‐treated booth to ensure participants had normal hearing. A modified Hughson‐Westlake procedure was used to measure pure tone thresholds from 0.125 to 8 kHz (0.125, 0.25, 0.5, 1, 2, 4, 6, and 8 kHz) in each ear of all participants. Speech discrimination scores were evaluated with phonetically balanced words at the most comfortable loudness level. Acoustic tympanometry tests were performed on all participants using a 226‐Hz probe tone.

#### 2.2.2 Emotional Prosody Recognition Assessment

**FIGURE 1:**
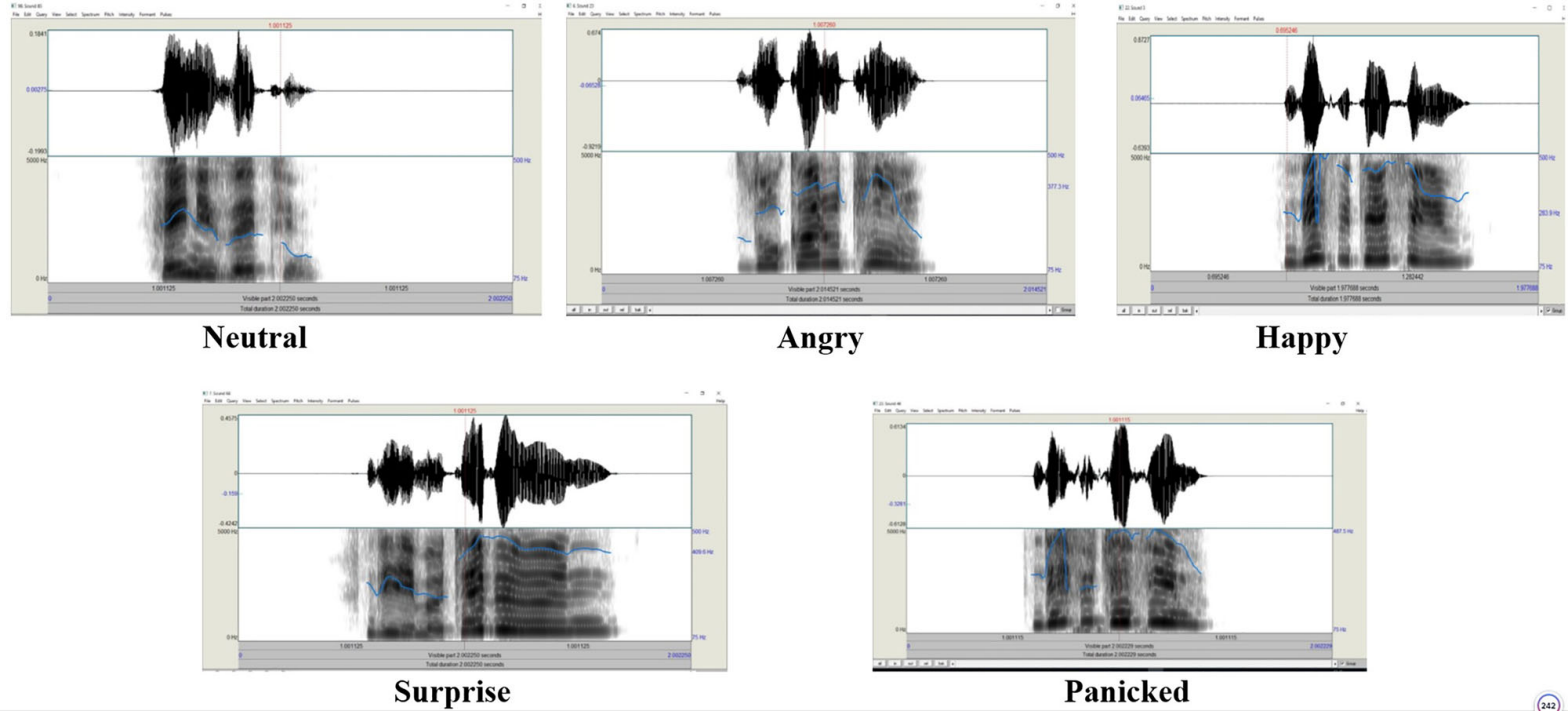
F0 contour of the five emotions voiced by a Turkish speaker.

Prosody was assessed by asking participants to identify five emotions including neutral, angry, happy, surprise, and panic. A one interval five alternative forced‐choice methodology was used in which users had to choose one of the five response options for each auditory stimulus presented. Twelve nonlinguistic three‐syllable verbal words were created (two for practice) in the order of Consonant‐Vowel‐Consonant (CVC), Vowel‐Consonant‐Vowel (VCV), and Consonant‐Vowel‐Consonant (CVC), for example, “fel ili sem” [fɛl ili sɛm]. The pseudo‐speech stimuli were nonmeaningful for both English and Turkish, as confirmed by adjudicators (an audiologist, a psychologist, a linguist, nonprofessionals, and a speech therapist). After the practice phase, a hundred stimuli, including 5 emotions × 2 speakers × 10 pseudowords, were presented to the participants. Mean fundamental frequency (F0), F1, F2, and F3 contours for each sentence created with each target emotion and pitch counter of the emotions were measured using Praat (www.praat.org), as shown in Figure 1 and Table 1. All stimuli were recorded by male and female native Turkish speakers in a studio. Therefore, we were able to determine if speaker language dominance influences the validity of pseudo‐sentences. Speakers have at least 5 years of experience as an announcer and a thespian without any speech, language, or hearing disorders. Speakers were informed about voicing five emotions with an imaginary scenario without overacting.

**TABLE 1:** Mean fundamental and three formant frequencies of the recorded stimulus.

	Male				Female			
Emotions	F0	F1	F2	F3	F0	F1	F2	F3
Neutral	145.5	572.0	1817.2	3022.1	252.0	554.3	1772.6	2984.5
Angry	187.0	577.0	1788.0	2966.8	281.6	586.2	1786.1	2966.1
Panic	232.9	629.1	1729.0	2965.8	403.1	618.0	1729.7	2965.0
Surprise	252.5	604.7	1694.3	2899.6	376.6	604.4	1690.8	2899.5
Happy	304.7	606.1	1667.9	2644.7	390.4	607.7	1717.5	2921.9

The C# programming language (Microsoft) was used to deliver the pseudo‐speech stimuli to the participants. The stimuli were presented randomly. The percentage of correct answers for each emotion, the average responses for all emotions, and the reaction time for correct answers were all calculated. Participants sat comfortably, wore supraural headphones, and used the mouse to select emotions on the computer screen. The stimuli were presented bilaterally at 40 dB SL ± 3 dB SPL with a max 2‐s duration. The computer displayed all emotions at the same distance from the center box. The mouse cursor automatically returned to the center box after each selection. Emotional prosody and reaction time of the correct answers during emotional prosody assessment were analyzed between two groups.

#### 2.2.3 Motor Response Time Assessment

To account for response time using the computer mouse to select responses during the test, motor response time was calculated due to differences in upper extremity coordination performance or mouse familiarity. The purpose of this test was to see how fast the participants could use the mouse. We planned a digit recognition test that was independent of linguistic features for this purpose. Random digits from 1 to 5 were presented, which ensured low cognitive load without much linguistic information. Each digit was repeated five times (25 auditory stimuli). The numbers were presented to the participants using the C# programming language (Microsoft) as the same procedure as the emotional prosody perception assessment.

#### 2.2.4 Reaction Time Assessment

To understand the group differences, we assessed the reaction time of the correct emotional response during prosody recognition. During the emotional prosody perception test the program automatically recorded the time between the stimulus presentation and the answers of the participants. Only correct answers’ reaction times were analyzed. Participants were not made aware that reaction times were being assessed, and the instructions focused on correctness.

### 2.3 Data Analyses

The IBM SPSS Statistics software version 23 (IBM Corp., Armonk, NY) was used for all the statistical analyses. The assumption of normal distribution was assessed using visual (histograms, probability plots) and analytical (Kolmogorov–Smirnov test) methods. Descriptive analyses were presented using means and standard deviations. As the data met the assumption of normality, F0 differences in terms of talkers’ sex and emotions were compared using two‐way repeated measures ANOVA. Group differences were compared using an independent samples *t*‐test. We performed multiple pairwise comparisons, thus, the Type‐I error level was adjusted to *p* < 0.01 using the Bonferroni correction. Effect sizes were calculated using Cohen's *d*, defined as (*M*_2_ − *M*_1_) ⁄ √((*SD*_1_^2^ + *SD*_2_^2^) ⁄ 2).

## 3 Results

### 3.1 Participants

A total of 30 American participants with a mean age of 22.78 ± 2.71 and 30 Turkish participants of the same sex, age, and education level were included in the study (see Table 2). The mean pure tone average (PTA) was 5.73 dB HL (Right) and 6.03 dB HL (Left) for American participants, and 5.66 dB HL (Right) and 5.96 dB HL (Left) for Turkish participants, confirming no difference in hearing sensitivity between groups.

**TABLE 2:** Demographic information of the participants.

	Turkish (*n* = 30)			American (*n* = 30)		
	Female (*n* = 15)	Male (*n* = 15)	All	Female (*n* = 15)	Male (*n* = 15)	All
Age in years (Mean ± SD)	23.44–2.25	21.93–3.10	22.73–0.5	23.67–2.26	22.00–3.00	22.83–0.5
Right ear PTA (Mean ± SD)	6.44–2.68	4.79–2.75	5.66–0.51	5.20–4.13	6.27–4.37	5.73–0.76
Left ear PTA (Mean ± SD)	6.56–2.50	5.29–2.81	5.96–0.49	4.80–4.80	7.27–4.28	6.03–0.84
Education (N)	Undergraduate: 19 Graduate: 10 Master's degree: 1			Undergraduate: 19 Graduate: 10 Master's degree: 1		

Abbreviations: N, number; PTA, pure tone average from 0.5 to 4 kHz; SD, standard deviation.

### 3.2 Acoustic Analyses of the Stimulus

**FIGURE 2:**
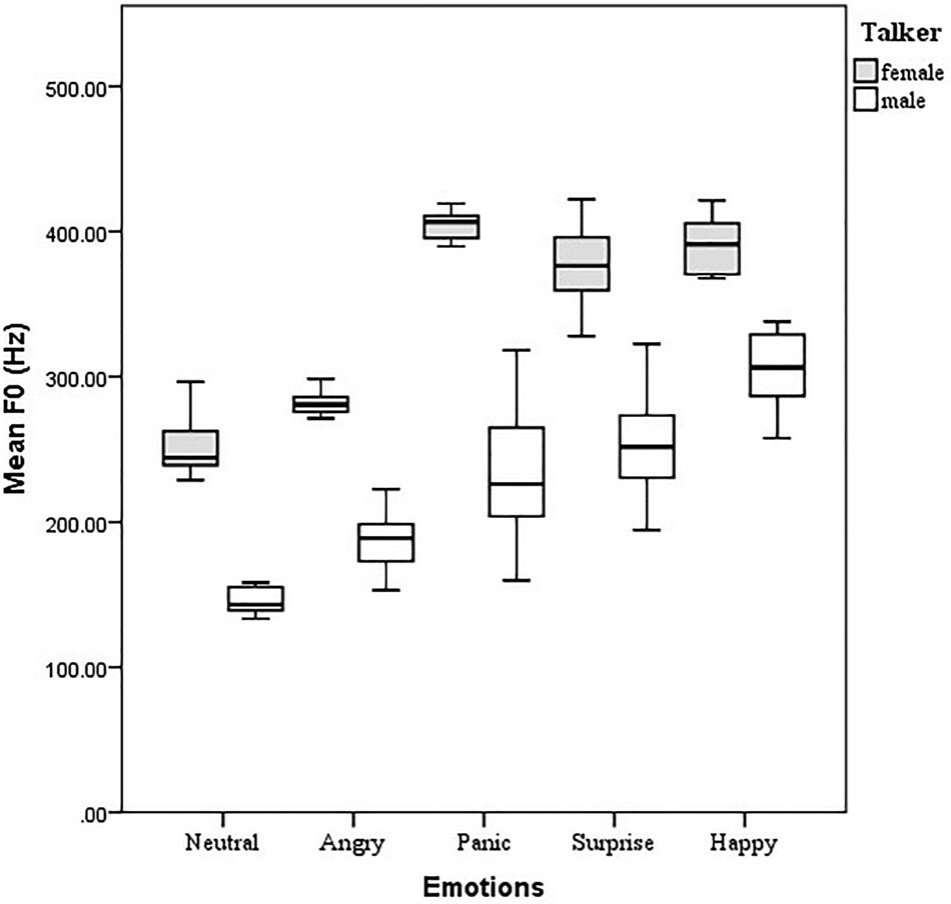
Mean F0 values of the pseudo‐sentences for the five target emotions recorded by a male and female talker.

The results of a two‐way repeated measures analysis of variance revealed that there was a statistically significant interaction between the effects of emotions and talkers (*F* (4, 95) = 7.448, *p* < 0.001) on the F0 value. The mean F0 values significantly varied between the two talkers *F* (1, 98) = 105.4, *p* < 0.001) and among the five target emotions *F* (4, 95) = 105.4, *p* < 0.001). The post hoc Tukey test showed that for the male and female talkers, target emotions were ordered in terms of mean F0 values (from high to low) as happy, surprise, panic, angry, and neutral. The mean F0 values were significantly different between all target emotions, as seen in Figure 2.

### 3.3 Comparison of Emotional Recognition Between Groups

**FIGURE 3:**
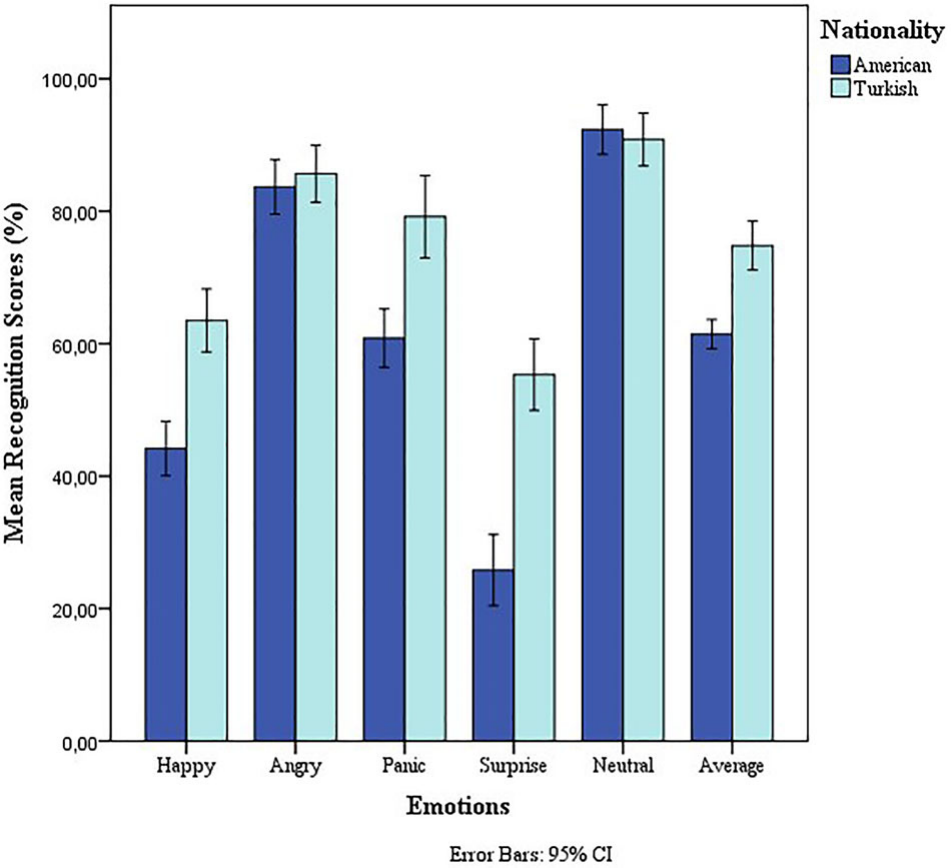
Comparison of the emotional conditions between two groups. Error bars indicate a 95% confidence interval.

The emotional recognition scores and average emotion scores between the groups are presented in Table 3 and Figure 3. Surprise was the least recognizable emotion whereas angry and neutral were the most easily recognizable emotions by participants from both groups. There was no statistically significant difference in recognition of neutral and angry between the groups. However, there was a statistically significant difference in recognition of the remaining three emotions (happy, surprise, and panic) between the groups. There was also a statistically significant difference observed in the overall average scores (*p* < 0.001). The average score of the five different emotional conditions showed that Turkish participants showed higher recognition scores in the pseudo‐speech corpus than the English‐speaking participants. This showed the recorded materials' language dominance effect, although stimuli were pseudo‐sentences for each group.

**TABLE 3:** Recognition of the emotions in Turkish and American participants.

Emotions	American		Turkish		*t*	df	*p*	Cohen's *d*
	Mean (SD)	Min–Max	Mean (SD)	Min–Max				
Neutral (%)	92.3 (1.8)	55.0–100.0	91.5 (1.6)	75.0–100.0	0.5	58	0.7	0.4
Angry (%)	83.7 (2.0)	60.0–100.0	85.6 (2.1)	55.0–100.0	−0.6	58	0.5	0.9
Happy (%)	44.2 (2.0)	20.0–65.0	63.5 (2.3)	40.0–85.0	−6.2	58	< 0.001^*^	8.9
Surprise (%)	25.8 (2.6)	5.0–50.0	55.6 (2.7)	15.0–75.0	−7.9	58	< 0.001^*^	11.2
Panic (%)	60.8 (2.1)	35.0–80.0	78.0 (3.1)	40.0–100.0	−4.5	58	< 0.001^*^	6.4
Average (%)	61.4 (1.1)	49.0–74.0	74.9 (1.7)	53.0–87.0	−6.3	47.5	< 0.001^*^	9.4

Abbreviation: SD, standard deviation.

* *p* < 0.01

### 3.4 Comparison of Motor Response Time and the Reaction Time Between Groups

**FIGURE 4:**
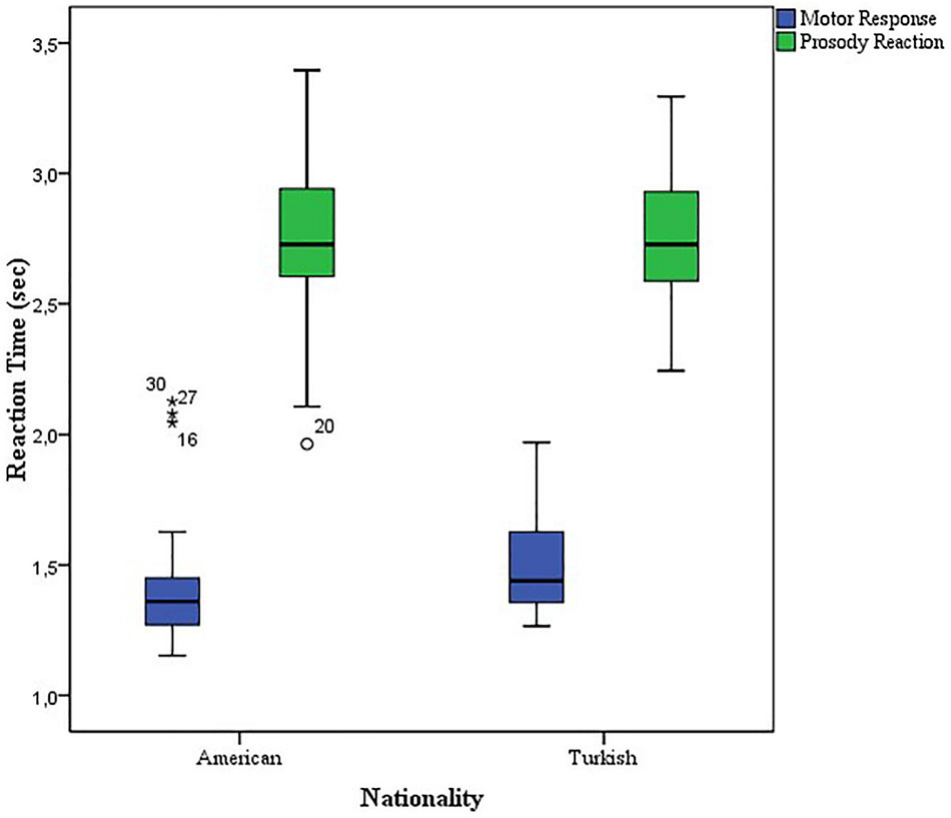
Motor response time and reaction time of the emotional prosody stimuli between two groups.

The mean motor response time was 1.43 (SD = 0.04; min–max = 1.13–2.11) and 1.51 (SD = 0.03; min–max = 1.27–1.97) s for American and Turkish participants, respectively. There were no statistically significant differences in response time between the two groups, according to the independent samples *t*‐test [*t* (58) = −1.483, *p* = 0.14] as shown in Figure 4. The effect size was 2.8. Therefore, in interpreting the results for reaction time, we exclude the effect of familiarity with computer mouse use.

The mean reaction times were 2.75 (SD = 0.06; min–max = 1.96–3.40) and 2.74 (SD = 0.04; min–max = 2.24–3.30) s for American and Turkish participants, respectively. According to the independent sample *t*‐tests, there were no significant differences between the two groups [*t* (58) = 0.097, *p* = 0.92], as shown in Figure 4. The effect size was 0.1.

## 4 Discussion

The current study aimed to assess emotional recognition ability using corpora of emotions conveyed through nonlinguistic pseudo‐sentences recorded by Turkish speakers in native and non‐native listeners. The long‐term goal of this initiative is to see if it is possible to have a language‐neutral emotional perception test battery that can be used in different cultures and/or countries. Fundamental frequency (F0) was the primary acoustic parameter analyzed in this study, as it is the most critical cue for emotional prosody perception, widely supported by prior research (Juslin and Laukka 2003; Scherer 2003). While formant frequencies (F1, F2, and F3) were reported for reference, they primarily contribute to phonemic identity rather than prosodic modulation and were not central to the study's objectives. Given that pseudo‐sentences lack meaningful linguistic content, formant variations are expected to be less systematically related to emotional expression. The inclusion of these parameters provides a comprehensive acoustic profile of the stimuli and serves as a reference for future studies exploring additional spectral characteristics in emotional prosody perception.

Previous research suggests that anger is one of the most easily recognizable emotions due to its distinct acoustic features, high arousal, and negative valence (Juslin and Laukka 2003; Scherer 2003). Our findings align with this, as anger and neutral emotions were recognized with similar accuracy across both language groups, suggesting that these emotions may rely on universal prosodic cues that transcend linguistic boundaries.

However, while panic is also a high‐arousal, negative emotion, it did not exhibit the same pattern. This suggests that emotional recognition difficulty may not be solely determined by valence (positive vs. negative) or arousal (high vs. low) but rather by the specific acoustic features associated with each emotion. Panic may share prosodic characteristics with other emotions, making it more prone to confusion, whereas anger has a more distinct prosodic profile, with sharper pitch contours, greater intensity, and more abrupt changes in duration (Scherer 2003).

This finding highlights the complexity of cross‐linguistic emotional prosody perception, suggesting that not all negative emotions are equally recognized, and language dominance effects may be more pronounced for some emotions than others. Future research should further investigate the role of acoustic distinctiveness in shaping crosslinguistic emotional recognition.

In daily communication, emotions are expressed with varying intensities, which adds to the difficulty in recognizing them. For example, anger can appear as “cold anger,” expressed in a controlled way, or “hot anger,” with more intense expression. Recognizing these variations depends on interpreting acoustic cues, such as pitch changes and intensity patterns. Low‐frequency sounds provide important prosodic information for emotional communication, while high‐frequency sounds help convey verbal content (Scherer 2003).

For example, neutral utterances tend to lose intensity toward the end of the sentence. In contrast, anger maintains a steady or increased intensity throughout. Happiness, on the other hand, often begins with lower intensity, peaks in the middle, and tapers off toward the end. These intensity patterns help distinguish emotions, with higher intensity often linked to anger, lower intensity to neutral expressions, and rising intensity to happiness (Ćwiek et al. 2021; Castro and Lima 2010). These patterns may be similar across cultures, aiding the recognition of angry and neutral emotions across different groups, irrespective of semantics.

Other aspects, such as pitch patterns, duration, and speech rate, also contribute to emotional recognition (Castro and Lima 2010). Together, these features may form a foundation for interpreting emotions, even in diverse cultural or linguistic contexts.

A previous study on emotion recognition in the context of cultural differences found no cross‐cultural effects between Dutch and English speakers, even though the stimuli were produced by Dutch native speakers (Nagels et al. 2019). However, this study was limited to only three core emotions (happiness, anger, and sadness), and the languages compared belonged to the same Germanic branch of the Indo‐European language family (Roberge 2020). Our findings partially align with this study but also reveal notable differences. This discrepancy may be explained by the proximity of the two emotions (happiness and surprise) in our recordings. Surprise, a common emotion in daily life, has characteristics that remain ambiguous, particularly regarding its valence (Noordewier and Breugelmans 2013). Research suggests that surprise can be either positive or negative, depending on whether the event aligns with an individual's goals (Noordewier and Breugelmans 2013). In our study, surprise was recorded in a positive state, making it acoustically similar to happiness when expressed in a positive manner. This overlap may account for the differences in findings between the previous study and ours regarding happiness.

Based on these results, we propose that when emotions are not accompanied by others with closely related characteristics, there may be no cross‐cultural effects in emotion recognition, even between languages as distinct as Turkish and English. In addition, there are some logical rationales why no difference was seen between different cultures. For instance, Jürgens et al. (2013) suggested that “fear” has no cultural differences. Altrov (2013) suggested that “sadness” could be universal while “joy,” “anger,” and “neutrality” are culture‐specific manner. On the contrary, we revealed that “anger” is universal, which is compatible with a previous study that found “anger” and “sadness” are more accurately recognized in five different languages (Thompson and Balkwill 2006). This could be explained by the evolutionary hypothesis that some emotions are more sensitive than others based on survival against threats.

Reaction time is well known to relate to cognitive load or listening effort (Giuliani et al. 2021). Since participants used a PC mouse, before reaction time assessment, motor response time with minimal cognitive load was assessed. Participants showed no differences. This means the reaction time of emotional stimuli showed cognitive loads for two different groups accurately. However, we found no group differences in terms of processing emotional stimuli. Listening to foreign languages and being exposed to different degrees of acoustic mismatch requires varying levels of cognitive recruitment (Peelle 2018). Pseudo‐sentences result in the same listening efforts for two cultural groups even though recorded with Turkish speakers. This can show that there is no native speaker language dominance in the processing of pseudo‐sentences. Reaction time assessment could be a way to reveal how irrespective pseudo‐sentences are from the native language of the participants. If participants are asked to focus on accuracy or response speed, reaction time tests may generate different results (Christensen et al. 2019). The instructions in the current study emphasized precision; participants were unaware that their reaction times were being measured.

### 4.1 Study Limitations and Future Directions

We recorded the utterances from professional speakers with imaginary feelings; however utterances could have been recorded by nonprofessional speakers as well or selected from real‐life recordings in broadcasts or social videos. Another vocal stimulus is synthetic speech without human speech recordings (Schröder 2001). With synthetic stimuli, researchers might easily control the acoustic parameters of the stimuli and perform well‐controlled studies. Although synthetic stimuli appear to be unnatural and “robot‐like,” to create vocal stimuli without culture and linguistic features, further studies should focus on this. All the previous studies, including ours, used forced‐choice methodology. This could result in artificially higher recognition rates. We used pseudo linguistic stimuli; future works could report nonlinguistic vocal stimuli to develop the universal auditory prosody test battery.

## 5 Conclusion

Overall results suggest that while there may be recognizable vocal cues irrespective of languages for expressing angry and neutral emotions, this does not apply to all emotions. These results point to the fact that the type of test materials may play an important role when measuring emotional recognition among different cultures using auditory stimuli. Reaction time evaluation can be employed to gauge the disparity between pseudo‐sentences and the participants' native language, as well as to assess the participants' listening effort during affective prosody evaluation. In terms of reaction time results pseudo‐sentences could be used for cross‐language auditory emotion recognition, however, with certain emotions. A full list of the pseudosentences used in this study is provided in the supporting information.Supporting Information

## Author Contributions

**Emre Gürses**: conceptualization, methodology, investigation, writing–original draft, software, formal analysis, data curation, writing–review and editing. **Sıdıka Cesur**: conceptualization, writing–review and editing, data curation, writing–original draft. **Vinaya Manchaiah**: writing–review and editing, supervision, data curation, methodology.

## Ethics Statement

All procedures performed in studies involving human participants were in accordance with the ethical standards of the institutional and national research committee and with the 1964 Helsinki Declaration and its later amendments or comparable ethical standards.

## Consent

Informed consent was obtained from all individual participants included in the study.

## Conflicts of Interest

The authors declare no conflicts of interest.

### Peer Review

The peer review history for this article is available at https://publons.com/publon/10.1002/brb3.70475.

## Supporting information



Supporting Information

## Data Availability

The datasets generated during and/or analyzed during the current study are available from the corresponding author.
