# Supplementary material for: Auditory Emotional Prosody Perception Using Pseudo‐Speech Stimuli in Native and Non‐Native Listeners
Source: Brain Behav. 2025 Apr 7;15(4):e70475. doi: 10.1002/brb3.70475 (PMC11975632; doi:10.1002/brb3.70475)
Supplement: Supplementary file 1 — Supporting Information [file BRB3-15-e70475-s001.docx]

The corpuses used in the study

- /tʌn ˈɑː.fə bʌn/
- /fɛl ˈɪ.li sɛm/
- /zæk ˈɑː.rə tʌk/
- /dɛk ˈɛ.ki pʊk/
- /dɛf ˈjuː.tɛ fɪm/
- /zɒk ˈɑː.ruː tʌm/
- /lʌn ˈɑː.vu sæv/
- /hɛn ˈaɪz tɪn/
- /ʃɛm ˈjuː.keɪ sɜm/
- /bæp ˈɑː.zu jɪt/
- /ˈuː.jə tɒs ˈɑː.və/
- /dɪm ˈɜː.tə vʌn**/**
